# Multi-omics analysis of the regulatory effects of low-phosphorus stress on phosphorus transport in soybean roots

**DOI:** 10.3389/fpls.2022.992036

**Published:** 2022-09-02

**Authors:** Hongyu Li, Letian Xu, Jiaxin Li, Xiaochen Lyu, Sha Li, Chang Wang, Xuelai Wang, Chunmei Ma, Chao Yan

**Affiliations:** ^1^College of Agriculture, Northeast Agricultural University, Harbin, China; ^2^College of Resources and Environment, Northeast Agricultural University, Harbin, China

**Keywords:** low phosphorus stress, phosphorus transport, transcriptomics, proteomics, metabonomics

## Abstract

The regulatory effects of uneven phosphorus supplies on phosphorus transport in soybean roots are still unclear. To further analyze the regulatory effects of low-phosphorus stress on phosphorus transport in soybean roots and the effects of uneven phosphorus application on the physiological mechanism of phosphorus transport in soybean roots, dual-root soybean plants were prepared via grafting, and a sand culture experiment was performed. From the unfolded cotyledon stage to the initial flowering stage, one side of each dual-root soybean system was irrigated with a low-phosphorus-concentration solution (phosphorus-application [P+] side), and the other side was irrigated with a phosphorus-free nutrient solution (phosphorus-free [P-] side); this setup allowed the study of the effects of different phosphorus supply levels on the expression of genes and proteins and the accumulation of metabolites in soybean roots on the P- side to clarify the method through which phosphorus transport is regulated in soybean roots and to provide a theoretical basis for improving the use rate of phosphorus fertilizer. The results revealed that the unilateral supply of low-concentration phosphorus promoted the uptake of phosphorus by soybean roots and the transport of phosphorus from the P+ side to the P- side. Compared with the normal concentration of phosphorus supply and the phosphorus-free supply, the low concentration phosphorus supply affected the regulation of the metabolic pathways involved in starch and sucrose metabolism, glycolysis, fructose, and mannose metabolism, etc., thereby affecting soybean root phosphorus transport. The low-phosphorus stress inhibited fructose synthesis and sucrose synthase synthesis in the soybean roots and the synthesis of hexokinase (HK) and fructose kinase, which catalyzes the conversion of fructose to fructose-6-phosphate. Low-phosphorus stress promoted the synthesis of sucrose invertase and the conversion of sucrose into maltose by the activity of starch synthase (StS) and stimulated the synthesis of UDPG pyrophosphorylase (UGP) and phosphoglucose isomerase (GP1), which is involved in the conversion of UDP-glucose to glucose-6-phosphate. The phosphorus transport pathway of soybean roots was then affected, which promoted phosphorus allocation to UTP and glucose-6-phosphate. Additionally, low-phosphorus stress hastened glycolysis in the soybean roots and inhibited the synthesis of malic acid, thereby promoting the transport of phosphorus in the roots. In addition, low-phosphorus stress inhibited the synthesis of fructose, mannose, and mannose-1-phosphate and the synthesis of other enzymes involved in phosphorus transport as well as invertase, thereby inhibiting the transport and synthesis of several organic phosphorus-containing compounds.

## Introduction

Soybean (*Glycine max* L.) is an important grain and oil crop species in China (Messina, 1999). Phosphorus is an important nutrient for soybean growth, and phosphorus deficiency inhibits soybean growth and metabolism (Chen et al., 2011; Qin et al., 2011, 2012; Li et al., 2015). Due to the different methods of tillage and fertilization, the distribution of phosphate fertilizer in the soil is uneven; as such, some root hairs and epidermal cells can obtain sufficient phosphate fertilizer amounts to meet their needs, while other cannot. Therefore, some roots of soybean are often under low-phosphorus stress, which affects plants growth and yield, especially in the middle and late growth stages (Raghothama and Karthikeyan, 2005). In addition, due to soil moisture, temperature and other factors, some phosphorus fertilizers compounds are unavailable to plants, and the mobility of phosphorus fertilizer in the soil is very poor, resulting in a decrease in the use rate of phosphorus by soybean plants. Therefore, analyzing the regulatory effects of different phosphorus supply levels on phosphorus transport in soybean roots is highly important. Therefore, it is highly important to analyze the regulatory effects of uneven phosphorus supplies on phosphorus transport in soybean roots.

In soybean, phosphorus mainly is obtained by the absorption of inorganic phosphorus (Pi) by the roots, which is subsequently translocated to the shoots for participation in cell synthesis, photosynthesis, respiration and other metabolic processes. Subsequently, secondary signals such as sucrose, phosphatase and mRNA are transmitted to the roots, resulting in a root feedback effect (Natr, 1992; Sakano et al., 1992). Soybean roots can regulate phosphorus uptake through physiological metabolic responses to adapt to low-phosphorus stress (Theodorou and Plaxton, 1993; Filippelli, 2008). Low-phosphorus stress can promote soybean root production to expand the phosphorus absorption area and increase the absorption of phosphorus, and low-phosphorus stress can also stimulate plants to secrete organic acids into the rhizosphere and to convert unavailable forms of phosphorus in the soil to plant-available forms of phosphorus to improve the use rate of phosphorus (Wang et al., 2010; Li et al., 2012). In recent years, with the development of the proteomic, metabolomic and genomic techniques, new knowledge has been gained about the metabolic and physiological processes associated with phosphorus absorption and transport in soybean roots under low-phosphorus stress. Proteomic analysis showed that the differentially expressed proteins (DEPs) in soybean roots under low-phosphorus stress are mainly involved in metabolic pathways such as “carbohydrate metabolism,” “energy metabolism,” “nucleotide metabolism,” and “signal transduction” (Sha et al., 2016; Zhao et al., 2021). The DEPs mainly include malic dehydrogenase, phosphoglucomutase, phosphoglyceric acid mutase, fructose kinase, and phosphoglyceric acid kinase (Vengavasi et al., 2017). The effects of low-phosphorus stress on phosphorylation within soybean roots were analyzed by metabolic and transcriptome analysis. It was found that the metabolic pathways involved in soybean root resistance to low-phosphorus stress include “phosphorylated metabolites,” “phosphorylated lipids and nucleic acids” and other metabolic pathways (Mo et al., 2019). Multiple genes involved in phosphorus uptake and transport in soybean roots have been identified (Tamura et al., 2012), and low-phosphorus stress was found to induce both the expression of the low-affinity Pi transporters *GmPT1* and *GmPT2* in soybean roots (Wu et al., 2011; Song et al., 2014) and the expression of the high-affinity Pi transporters *GmPT5* and *GmPT7* (Qin et al., 2012; Inoue et al., 2014), thereby promoting phosphorus uptake. In addition, it was found that low-phosphorus stress can induce the expression of the β-expansin gene *GmEXPB*_2_ and the root acid phosphatase gene *GmPAP* in soybean roots, which, together, can improve phosphorus absorption and utilization by regulating root architecture (Guo et al., 2008; Li et al., 2012).

In summary, in-depth studies on the absorption and transport of soybean phosphorus under low-phosphorus stress have been performed, but research on the regulatory effects of uneven phosphorus supplies on soybean root phosphorus transport is still scarce. Additionally, the existing research on phosphorus uptake by and transport in soybean roots under low-phosphorus stress has included mainly physiological and individual omic analysis. However, integrated multi-omic data analysis has become a popular method for use in systems biology research. Therefore, in this study, the method of dual-root soybean plants was used. A combined analysis was performed to study the regulatory effects of uneven phosphorus supplies on the metabolic pathway involved in phosphorus transport in soybean roots to provide a theoretical reference for analyzing the physiological mechanism underlying effects of phosphorus application on phosphorus transport in soybean plants.

## Materials and methods

This experiment was carried out at the experimental station of Northeast Agricultural University in Harbin city, Heilongjiang Province (126°43′ E, 45°44′ N), in 2021, and sand-cultured double-root soybean systems were used as research materials. The tested soybean (*Glycine max* L.) variety was Kenfeng 16, which was obtained from Heilongjiang Academy of Land Agricultural Reclamation Science, Heilongjiang, China. Dual-root soybean plants were prepared according to the grafting method described by Xia et al. (2017). The specific grafting method is shown in Supplementary Figure 1.

### Experimental treatments

From the VC stage (unfolded cotyledon stage) to the R1 stage (initial flowering stage), phosphorus-containing nutrient solution was poured onto one side of the dual-root soybean plants, which was considered the phosphorus-application (P+) side, and phosphorus-free nutrient solution was poured onto the other side, which was considered the P- side. Two different concentrations of phosphorus were applied on the P+ side, namely, 1 and 31 mg/L, and the corresponding treatments were denoted as LP and OP, respectively. In the experiment, KH_2_PO_4_ was used as the phosphorus source, and the nutrient solution was prepared as proposed by Hoagland and Arnon (1950) and Yao et al. (2018) (Supplementary Tables 1, 2). The potassium level of the LP treatment was adjusted to the same as that of OP treatment. From the VE stage (emergence stage) to the VC stage (unfolded cotyledon stage), the plants received 250 mL of distilled water once a day on each side. Then, 250 mL of P-containing nutrient solution was applied on the P+ side, 250 mL of P-free nutrient solution was applied to the P− side, and the plants at the V4 stage to R1 stage were treated two times a day.

### Sampling method

Samples were taken at the R1 stage (initial flowering stage). The shoots were cut along the grafting site between 8:00 and 10:00 on a sunny day. The belowground roots on both sides were washed with distilled water to remove the sand. Nodules were removed from the soybean roots on the phosphorus-free side P- side and transferred to a −80°C freezer for subsequent multiomics and sucrose, fructose, mannose, malic acid and asparagine concentration analysis. The test was repeated eight times for sucrose, fructose, mannose, malic acid, and asparagine concentration analysis, three times for transcriptomics analysis, three times for proteomic analysis, and eight times for metabolomic analysis.

### Test methods

#### Physiological index determination

The phosphorus concentration in the plants was determined by the molybdenum antimony colorimetric method (Murphy and Riley, 1962). The concentration of inorganic phosphorus in the plants was determined by trichloroacetic acid extraction and the molybdenum blue method (Taussky and Shorr, 1953). The concentrations of sucrose, fructose, mannose, maltose, malic acid, and asparagine were determined via high-performance liquid chromatography (HPLC) (Zakharova et al., 2013).

#### Proteome determination

##### Protein extraction

One gram of sample tissue was added to liquid nitrogen for grinding, and the proteins were extracted by using SDT (4% w/v SDS, 100 mM Tris/HCl (pH 7.6), and 0.1 M dithiothreitol [DTT]) buffer, and then the protein was quantified by a BCA Protein Assay Kit (Bio-Rad, United States). The proteins from each sample were digested by trypsinization via a filter-assisted proteome preparation kit. The peptide was desalted through C_18_ cartridges and redissolved in 40 μL of 0.1% formic acid solution after lyophilization. Finally, the peptides were quantified by determining the optical density at 280 nm (OD280).

##### Tandem mass tag labeling and protein digestion

According to the manufacturer’s instructions (Thermo Fisher Scientific, United States), tandem mass tag (TMT) reagent was used to label 1 × 10^–4^ g peptide mixture for each sample. The labeled peptides were fractionated by a High-pH Reversed-Phase Peptide Fractionation Kit (Thermo Fisher Scientific, United States) by gradually increasing the gradient elution with acetonitrile.

Liquid chromatography–tandem mass spectroscopy (LC–MS/MS) analysis was performed on a Q Exactive mass spectrometer (Thermo Scientific) that was coupled to Easy nLC instrument (Proxeon Biosystems, now Thermo Fisher Scientific) for 60/90 min. The peptides were loaded onto a reversed-phase trap column (Thermo Scientific Acclaim PepMap100, 100 μm × 2 cm, nanoViper C_18_) connected to a C^18^ reversed-phase analytical Easy Column (10 cm long, 75 μm inner diameter, 3 μm resin; Thermo Scientific) in buffer A (0.1% formic acid) and separated with a linear gradient of buffer B (84% acetonitrile and 0.1% formic acid) at a flow rate of 300 nl/min controlled by IntelliFlow technology, as described by Lyu et al. (2022) and Wang et al. (2022). After chromatographic separation, the sample was analyzed on a Q Exactive Mass Spectrometer in positive ion mode; the detailed methods used for detection are provided in Supplementary method 2.

Proteome Discoverer 1.4 software with the MASCOT engine (version 2.2; Matrix Science, London, United Kingdom) was used for the identification and quantitation of proteins using the data for “*Glycine_max*” from the NCBI database.

#### Metabolomic determination

##### Metabolite extraction

After the sample was slowly thawed at 4°C, 50 mg was weighed into a precooled of methanol:acetonitrile:water mixture (2:2:1, v/v), vortexed, sonicated at low temperature for 30 min, and maintained at −20°C. The mixture was incubated at room temperature for 10 min and subsequently centrifuged at 14,000 × *g* for 20 min at 4°C. The supernatant was collected and dried under a vacuum. Then, 100 μL of acetonitrile aqueous solution (acetonitrile:water = 1:1, v/v) was added for mass spectrometry analysis, reconstituted, and vortexed at 4°C. The mixture was subsequently centrifuged at 14,000 × *g* for 15 min, and the supernatant was collected for injection and analysis according to the methods of Wang et al. (2022).

##### LC–MS analysis

Samples were separated by an Agilent 1,290 Infinity LC ultra-high-performance liquid chromatography (UHPLC) HILIC column. The column temperature was 25°C, the flow rate was 0.5 mL/min, and the injection volume was 2 μL. The mobile phase solution A consisted of water +25 mM ammonium acetate +25 mM ammonia; B, acetonitrile. The gradient elution program was as follows: 0–0.5 min, 95% B; 0.5–7 min, linear decrease in B from 95% to 65%; 7–8 min, linear decrease in B from 65 to 40%; 8–9 min, maintenance of B at 40%; 9–9.1 minutes, linear increase in B from 40 to 95%; and 9.1–12 min, maintenance of B at 95%. The samples were placed in a 4°C autosampler throughout the analysis. For quadrupole time-of-flight–mass spectrometry (Q-ToF-MS), an AB Triple TOF 6600 mass spectrometer was used for the acquisition of first- and second-stage mass spectra. With respect to the electrospray ionization (ESI) source after separation by HILIC, the conditions were as described in Supplementary method 2.

#### Transcriptome determination

##### RNA extraction

A 0.5 g sample was extracted by the TRIzol (Invitrogen, Carlsbad, CA, United States) method. Genomic DNA was removed using DNase I (TaKaRa). The RNA quality was then determined using a 2100 Bioanalyzer (Agilent, Santa Clara, CA, United States) and quantified using an ND-2000 Spectrophotometer (NanoDrop Technologies).

##### Library construction and sequencing

Paired-end libraries were constructed using an ABclonal mRNA-Sq-Lib Preparation Kit (ABclonal, China). Oligo (dT) magnetic beads were used to purify mRNA from 1 μg of total RNA. Cleavage was then performed using divalent cations in ABclonal first-strand synthesis reaction buffer. Subsequently, first-strand cDNA was synthesized using the mRNA fragment as a template in conjunction with random hexamer primers and reverse transcriptase (RNAseH), and then second-strand cDNA was synthesized using DNA polymerase I, RNAseH, buffer, and dNTPs. The synthesized double-stranded cDNA fragments were polyadenylated and ligated to prepare a paired-end library. The cDNA was subsequently purified with an AMPure XP system (Beckman Coulter, Beverly, MA, United States) and amplified via PCR with adapter-ligated cDNA and adapter primers. Finally, sequencing was performed with an Illumina NovaSeq 6000 instrument.

### Statistical analyses

#### Transcriptome analysis

The original image data files obtained during high-throughput sequencing were converted into original sequences (sequenced reads) by CASAVA base calling (base calling) analysis, stored in FASTQ (referred to as fq) file format, and then subjected to Gene Ontology (GO) annotation analysis and Kyoto Encyclopedia of Genes and Genomes (KEGG) annotation analysis. The specific method is described in Supplementary method 2.

#### Proteomic analysis

The basic data from mass spectrometry were input into Proteome Discoverer 1.4 and MASCOT (Matrix Science, London, United Kingdom; version 2.2) for library term identification and quantitative analysis. The specific method is described in Supplementary method 2.

#### Metabolomic analysis

The basic data were converted into mzXML format by ProteoWizard in Wiff format, and then peak alignment, retention time correction and peak area extraction were performed by XCMS software. The specific method is described in Supplementary method 2.

#### Multi-omics combined data analysis

Based on the KEGG annotation data, Fisher’s exact test and the respective differentially accumulates molecules revealed by the three different omic analysis procedures were used for KEGG pathway enrichment analysis.

## Results

### Effects of low-phosphorus stress on the phosphorus uptake by and transport in dual-root soybean plants

Table 1 shows that the concentrations of phosphorus and Pi in the roots of both sides of dual-root soybean under uneven phosphorus supplies were significantly different. On the P+ side of the LP treatment (1 mg/L), the phosphorus content of the OP treatment (31 mg/L) and the Pi content of the OP treatment (31 mg/L) were 35.00 and 25.00%, respectively, in response to 3.23% (1/31) phosphorus fertilizer supply; however, the P- side of the LP treatment presented a 63.63% phosphorus content in the OP treatment and a 50.00% inorganic phosphorus content. In the OP treatment, the phosphorus content on the phosphorus-application side (P+) side was significantly higher (*P* ≤ 0.01) than that on the phosphorus-free side (P-) side. P- side compared with P+ side, the concentrations of phosphorus and Pi of the roots in the LP treatment were 1.00 and 0.67 in the P-/P+ treatment, 0.55 and 0.33 in the OP treatment. This indicated that low-phosphorus stress promoted the uptake of phosphorus by soybean roots and the transport of phosphorus from the P+ side to the P- side.

**TABLE 1:** Phosphorus and inorganic phosphorus concentrations in the roots of dual-root soybean (%).

Treatments	Phosphorus		Inorganic phosphorus	
	P+	P−	P+	P−
LP	0.07 ± 0.01b	0.07 ± 0.00b	0.03 ± 0.006b	0.02 ± 0.001b
OP	0.20 ± 0.01a**	0.11 ± 0.01a	0.12 ± 0.007a**	0.04 ± 0.003a

Dual-root soybeans one side of phosphorus supplied is denoted as P+, the other side of non-phosphorus supplied is denoted as P−, values represent mean ± standard error (n = 6), vertical comparison, different lowercase letters indicate a significant difference of 5% among different treatments. **, 0.01 levels of significant difference between phosphorus supply side (P+) and non-phosphorus supply side (P−), the same below.

### Effects of low-phosphorus stress on the non-structural carbohydrate content of dual-root soybean plants

Table 2 shows that the non-structural carbohydrate content of dual-root soybean plants under uneven phosphorus supplies was significantly different. On the P+ side, the LP treatment (1 mg/L) presented a 56.63% sucrose content, a 61.02% fructose content, and a 53.85% starch content, in response to 3.23% (1/31) phosphorus fertilizer supply; however, the P- side of the LP treatment presented a 67.79% sucrose content, a 70.83% fructose content, and a 80.26% starch content. There was no significant difference in the concentrations of sucrose, fructose or starch between the P+ and the P- side in the LP treatment (*P* ≥ 0.01). In the OP treatment, the concentrations on the P+ side were significantly higher (*P* ≤ 0.01) than those on the P- side. P- side compared with P+ side, the concentrations of sucrose, fructose and starch in LP treatment root were 1.03, 0.94, and 0.97 P-/P+, 0.86, 0.81, and 0.65 in OP treatment. This indicated that low-phosphorus stress promoted the accumulation of sucrose, fructose and starch in the soybean roots and the transport of these carbohydrates from the P+ side to the P- side.

**TABLE 2:** Concentration of unstructured carbohydrates in the roots of both sides of dual-root soybeans (mg/g FW).

Treatments	Sucrose		Fructose		Starch	
	P+	P−	P+	P−	P+	P−
LP	3.50 ± 0.08^c^	3.62 ± 0.38^b^	0.36 ± 0.01^b^	0.34 ± 0.01^c^	0.63 ± 0.02^b^	0.61 ± 0.01^b^
OP	6.18 ± 0.02^b^**	5.34 ± 0.17^a^	0.59 ± 0.01^a^**	0.48 ± 0.01^a^	1.17 ± 0.02^a^**	0.76 ± 0.02^a^

Dual-root soybeans one side of phosphorus supplied is denoted as P+, the other side of non-phosphorus supplied is denoted as P−, values represent mean ± standard error (n = 6), vertical comparison, different lowercase letters indicate a significant difference of 5% among different treatments. **, 0.01 levels of significant difference between phosphorus supply side (P+) and non-phosphorus supply side (P−), the same below.

### Proteomic analysis of roots on the P- side of dual-root soybean plants

Tandem mass tag quantitative proteomics was used to analyze LP and OP roots (Table 3). A total of 5,237 proteins were identified, of which quantitative information was available for 4,918 proteins. A total of 211 DEPs were screened according to an expression greater than 1.2 or less than 0.83 and a *P*-value < 0.05; 128 proteins were increased, and 83 proteins were decreased (Supplementary Table 3).

**TABLE 3:** Differential protein identification results of phosphorus-free side of dual-root soybean roots.

Treatments	Upregulated protein	Downregulated protein	Total
LP vs. OP	128	83	211

**FIGURE 1:**
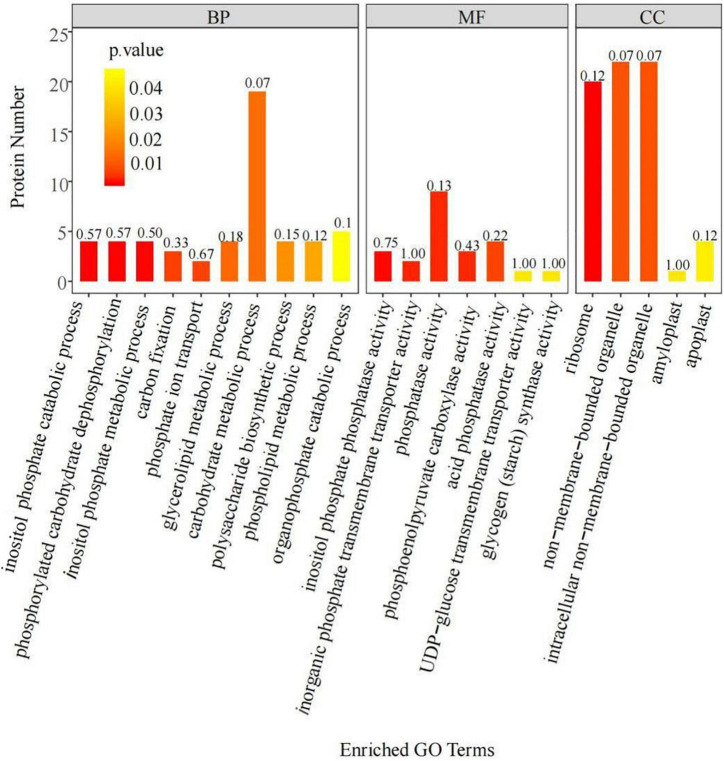
Gene Ontology (GO) annotation statistics of the differentially expressed proteins (DEPs) in soybean roots. The −log10 *P*-value, for each annotation.

Analysis of the DEPs identified above through BLAST alignments and GO annotations revealed the specific functions of the DEPs (Figure 1). According to their functional characteristics, the DEPs belonged to three groups: those related to cellular components (CCs), molecular functions (MFs), and biological processes (BPs). The DEPs in the CC group were mostly enriched in non-membrane bound organelles, intracellular non-membrane bound organelles, and ribosomes, among others. The DEPs in the MF group were mainly enriched in inositol phosphate phosphatase activity, inorganic phosphate transmembrane transporter activity, and phosphatase activity, among others. Those in the BP group were mostly enriched in carbohydrate metabolic processes, inositol phosphate catabolic processes, and phosphorylated carbohydrate dephosphorylation, among others. These results indicated that low-phosphorus stress could regulate phosphate transport and organophosphorus metabolism in soybean roots, ultimately affecting phosphorus transport.

**FIGURE 2:**
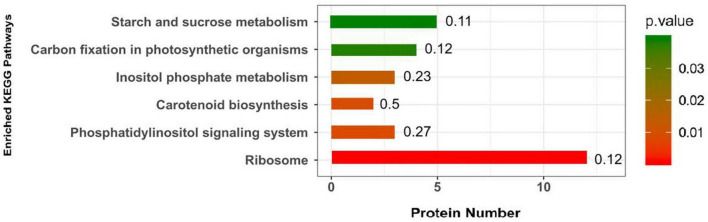
Kyoto Encyclopedia of Genes and Genomes (KEGG) pathway of the DEPs in soybean roots. The abscissa represents -log_10_ (*P-*value) for each pathway.

Kyoto Encyclopedia of Genes and Genomes pathways associated with DEPs were analyzed to determine the relevant metabolic or signaling pathways. As shown in Figure 2, the DEPs in the roots of the P- side of the dual-root soybean plants were mostly involved in ribosomes, phosphatidylinositol signaling system, carotenoid biosynthesis, inositol phosphate metabolism, carbon fixation in photosynthetic organisms, and starch and sucrose metabolism.

### Metabolomic analysis of the roots of the P- side of dual-root soybean plants

**FIGURE 3:**
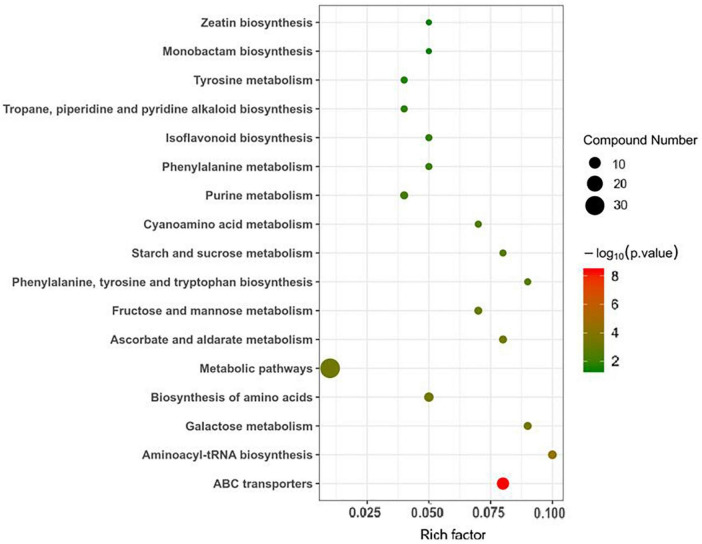
Metabolic pathways enriched with DEMs.

Ultra-high-performance liquid chromatography-Q-TOF MS was used to analyze the roots of soybean in the LP and OP treatments. Based on an OPLS-DA model with a VIP greater than 1 and a *P*–value < 0.05, 47 differentially expressed metabolites (DEMs) were identified, including 16 increased and 31 decreased DEMs (Supplementary Table 3). Figure 3 presents the metabolic pathways enriched with DEMs. The DEMs were enriched mostly in ABC transporters; aminoacyl-tRNA biosynthesis; galactose metabolism; biosynthesis of amino acids; metabolic pathways; ascorbate and aldarate metabolism; fructose and mannose metabolism; phenylalanine, tyrosine and tryptophan biosynthesis; starch and sucrose metabolism; cyanoamino acid metabolism; purine metabolism; phenylalanine metabolism; isoflavonoid biosynthesis tropane, piperidine and pyridine alkaloid biosynthesis; tyrosine metabolism; monobactam biosynthesis; and zeatin biosynthesis.

### Transcriptomic analysis of roots of the P- side of dual-root soybean plants

**FIGURE 4:**
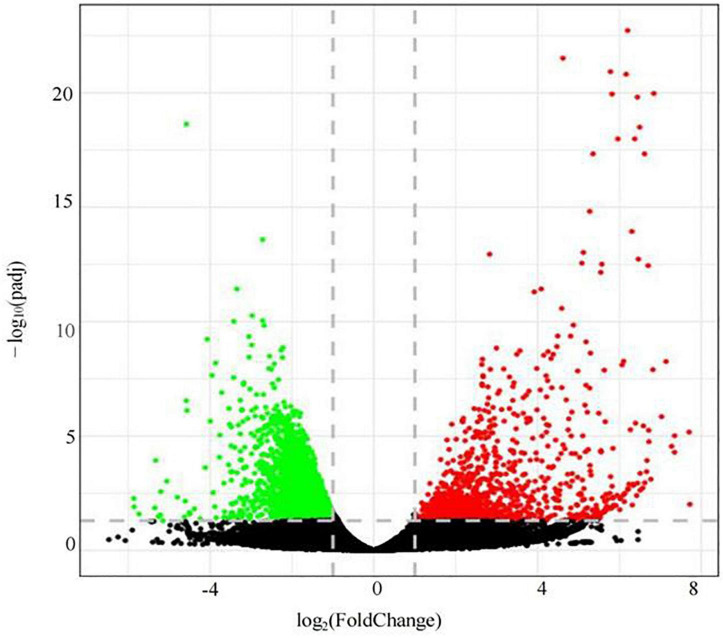
Differential gene volcano map.

Illumina high-throughput sequencing technology was used to analyze the roots of soybean in the LP and OP treatments (Figure 4). Based on *p*_adj_ < 0.05 and | log_2_(FC)| > 1, a total of 4,410 differentially expressed genes were screened, namely, 1,151 upregulated genes and 3,259 downregulated genes (Supplementary Table 4).

**FIGURE 5:**
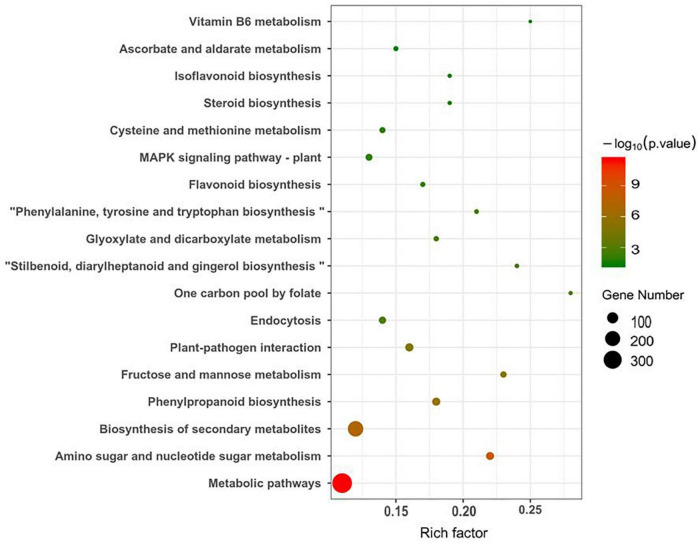
Differential gene KEGG bubble chart.

Figure 5 shows that the main KEGG pathways involvare metabolic pathways such as those involved in amino sugar and nucleotide sugar metabolism, biosynthesis of secondary metabolites, phenylpropanoid biosynthesis, fructose and mannose metabolism, and plant–pathogen interactions, as well as those involving one carbon pool by folate and one carbon pool by folate, among others.

### Multi-omicsic analysis of the roots of the P- side of dual-root soybean plants

**FIGURE 6:**
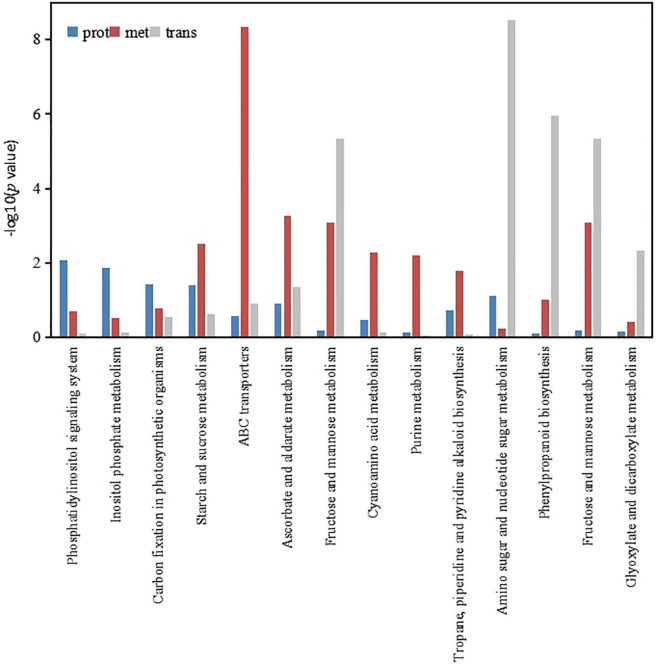
Histogram of significantly different protein, metabolism and gene KEGG enrichment.

According to the enrichment analysis results of the DEPs, DEMs and differentially expressed genes, a bar chart was constructed to show the degree of enrichment of the DEMs, DEPs and differentially expressed genes simultaneously. Figure 6 shows the KEGG pathways involved in these three omic findings, which included the following: those involved in phosphatidylinositol signaling system; inositol phosphate metabolism; carbon fixation in photosynthetic organisms; starch and sucrose metabolism; ABC transporters; ascorbate and aldarate metabolism; fructose and mannose metabolism; cyanoamino acid metabolism; purine metabolism; tropane, piperidine and pyridine alkaloid biosynthesis; amino sugar and nucleotide sugar metabolism; phenylpropanoid biosynthesis; fructose and mannose metabolism; and glyoxylate and dicarboxylate metabolism. These results indicated that low-phosphorus stress mainly regulated carbon metabolism and amino acid metabolism in soybean roots, thereby affecting phosphorus transport.

## Discussion

### Effects of low-phosphorus stress on the phosphorus uptake by and transport in soybean plants

Appropriate phosphorus level can promote phosphorus absorption and use in soybean (Zhang et al., 2014; Yao et al., 2018; Codling, 2019; Mo et al., 2022). In this study, it was found that the supply of low-concentration phosphorus (1 mg/L) resulted in 3.23% (1/31) of the normal phosphorus supply level (31 mg/L), which resulted in a phosphorus content of 46.51%, in dual-root soybean. The phosphorus content and 25% inorganic phosphorus content are different from the results found by Wang et al. (2021), who reported that, when soybean was supplied with 0.05 and 0.5 mmol/L KH_2_PO_4_, the 0.5 mmol/L treatment increased the root phosphorus efficiency. These results indicated that low-phosphorus stress promoted phosphorus uptake in soybean roots. This study also showed that the uneven supply of low concentrations of phosphorus promoted the transport of phosphorus from the P+ side to the P- side. These results are similar to the findings of Burleigh and Harrison (1999), Wouterlood et al. (2005), and Li et al. (2021), who used the split-root method to supply a higher concentration of phosphorus to one root of chickpea and soybean and a low concentration or no phosphorus to the other side, which promoted the absorption of phosphorus by the roots on both sides. In contrast to the use of the root separation method to apply phosphorus to only one side of *Lupinus albus*, Shu et al. (2007) found that phosphorus uptake in the roots was enhanced only on the P+ side and remained stable on the P- side. These results indicated that low-phosphorus stress stimulated the absorption and transport of phosphorus by soybean roots, thereby increasing the use rate of phosphorus.

### Effects of low-phosphorus stress on phosphorus transport signaling substances in soybean roots

**FIGURE 7:**
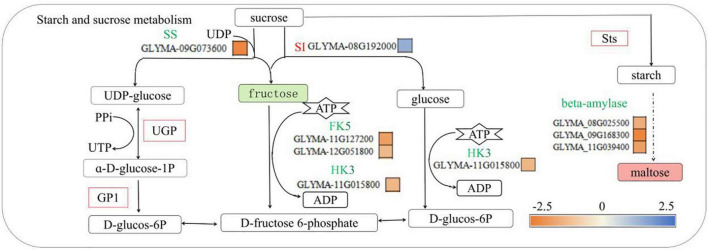
Starch and sucrose metabolic pathways involved in phosphorus transport in soybean roots. SI, sucrose transferase; SS, sucrose synthase; HK3, hexokinase 3, FK5, fructokinase 5, UGP-UDPG pyrophosphatase, Sts, starch synthase, UGP-UDPG pyrophosphorylase; bata- amylase, beta-amylase. Green solid squares indicate downregulated metabolites, red solid squares indicate upregulated metabolites, red fonts indicate upregulated genes, green fonts indicate downregulated genes, red squares indicate upregulated proteins, and yellow and blue squares indicate fold changes in gene expression.

Low-phosphorus stress can inhibit the photosynthesis of crops plants, promote the accumulation of sucrose and starch, and promote the transfer of sucrose as a signaling substance from the aboveground parts to the belowground parts, thereby stimulating the response effects of roots to low-phosphorus stress (Gibson, 2004; Morcuende et al., 2007; Muller et al., 2007). Sucrose is converted to starch under the action of starch synthase (StS) and can be further converted to maltose. In addition, sucrose is decomposed into UDP-glucose, fructose and glucose under the action of sucrase and invertase, and then under the action of UDPG pyrophosphatase (UGP), UDP-glucose reacts with pyrophosphate (PPi) to generate UTP. Glucose-6-phosphate, fructose and glucose are catalyzed by hexose kinase (HK3) and fructose kinase (FK5) to produce fructose-6-phosphate and glucose-6-phosphate, respectively, while ATP is consumed (Shivanna et al., 1989; Okazaki et al., 2009; Tang et al., 2016). In this study, there was no significant change in sucrose concentration in the P- side roots of soybean under low-phosphorus treatment, but with increasing maltose concentration, the fructose concentration decreased, the expression of the sucrose synthase *GLYMA-09G073600* gene decreased, the expression of the sucrose invertase *GLYMA-*08G192000 gene increased, and the expression of StS increased (Figure 7), and it is consistent with the physiological data in Table 2 and Supplementary Table 6. Low-phosphorus stress inhibited fructose synthesis and sucrose synthase synthesis in soybean roots but promoted sucrose invertase synthesis and sucrose conversion into maltose by StS, indicating that low-phosphorus stress affected the phosphorus transport pathway. These results are similar to those of Georgiev and Tsvetkova (2011) and Yang et al. (2020) who found that low-phosphorus stress had no significant effect on the accumulation of sucrose in soybean roots but promoted the accumulation of sucrose and starch in the leaves. While Garcia-Caparros et al. (2021) found that low-phosphorus stress reduced the contents of glucose, sucrose, and fructose in mung bean roots, as well as the activities of sucrose synthase and invertase, there was no significant difference in starch content, and Hermans et al. (2006) found that under short-term low-phosphorus stress, the accumulations of large amounts of starch, glucose, fructose and sucrose in the roots, stems and leaves of plants were different. It may be that the long-term low-phosphorus stress in this study caused the lack of phosphorus in the soybean roots and inhibited the metabolism of sucrose, thereby promoting the conversion of sucrose to maltose. This study also found that the expression levels of *GLYMA-11G15800*, *GLYMA-11G127200*, and *GLYMA-12G051800* were decreased, while the expression levels of UGP and phosphoglucose isomerase (GP1) were increased. These findings are the same as those of Rychter and Randall (1994), who concluded that the hexose kinase (HK) expression in pea roots decreased under low-phosphorus stress, but different from those of Vengavasi et al. (2017) who concluded that the expression of fructose kinase in soybean roots was upregulated under low-phosphorus stress. It may be that, in this study, long-term low-phosphorus stress reduced the fructokinase and hexokinase metabolic substrate ATP Gniazdowska and Rychter (2001), thereby inhibiting the activities of hexokinase and fructokinase, which catalyze the conversion of fructose to fructose-6-phosphate. Similar results were reported by Stitt (1998), who found that, under low-phosphorus stress, PPi can be an alternative energy donor of ATP involved in UGP synthesis from fructose-6-phosphate. This indicated that low-phosphorus stress inhibited the synthesis of HK and fructose kinase, which are involved in the conversion of fructose to fructose-6-phosphate in soybean roots, and stimulated the synthesis of UDPG pyrophosphorylase (UGP) and glucose phosphate isomerase (GP1), which convert UDP-glucose to glucose-6-phosphate, thereby promoting the conversion of phosphorus in soybean roots to UTP and to glucose-6-phosphate.

### Metabolic regulation of phosphorus transport energy in soybean roots under low-phosphorus stress

**FIGURE 8:**
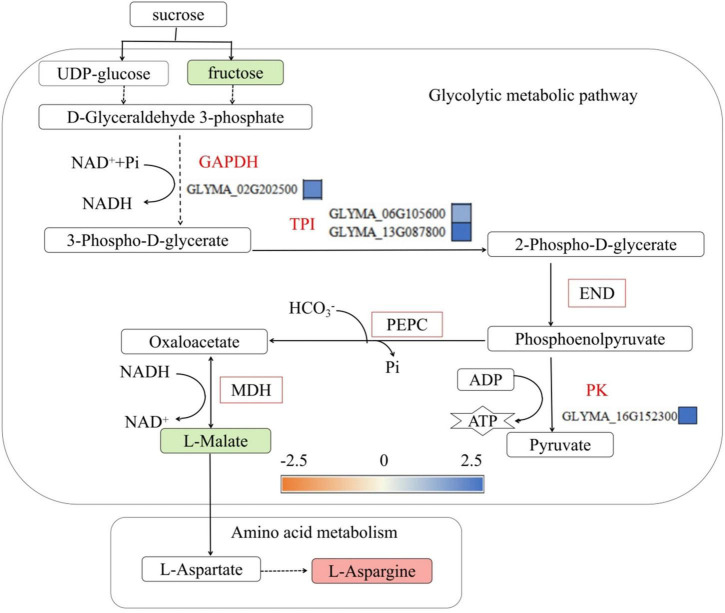
Glycolytic metabolic pathways involved in phosphorus transport in soybean roots. FBA, fructose 1,6-bisphosphate aldolase; GAPDH, glyceraldehyde-3-phosphate dehydrogenase; TPI, phosphoglycerate isomerase; END, Enolase; PEPC, phosphoenolpyruvate carboxylase; MDH, malate dehydrogenase. The green solid squares indicate downregulated metabolites, red solid squares indicate upregulated metabolites, red font indicates upregulated genes, green font indicates downregulated Genes, red squares represent upregulated proteins, and yellow and blue squares represent fold changes in gene expression.

Inorganic phosphorus and ATP participate in glycolysis, ensure that energy supplies are sufficient and participate in carbon skeleton formation. The glycolysis process begins with sucrose, and sucrose decomposes into fructose and glucose under the action of sucrose synthase, after which it is converted to malic acid under the action of a series of enzymes (Tripodi and Podestá, 2003). Therefore, the glycolytic metabolic process is affected by phosphorus transport in plants. This study found that low-phosphorus treatment decreased the malic acid concentration in the roots of the P- side of soybean, it is consistent with the physiological data in Supplementary Table 6, and the genes encoding glyceraldehyde-3-phosphate dehydrogenase (GAPDH) (*GLYMA-02G202500*) and triose phosphate isomerase (TPI) in the glycolytic metabolic pathway (*GLYMA-06G105600* and *GLYMA-13G087800*), enolase (END), phosphoenolpyruvate carboxylase (PEPC) and malate dehydrogenase (MDH) were upregulated (Figure 8). Upregulation of glycolytic enzymes increases ATP production and accelerates phosphorus metabolism and transport. This was also reported by Stevens et al. (2019), who found that low-phosphorus stress increased the activities of PEPC, MDH and other enzymes in soybean roots but inhibited the accumulation of malic acid, and by Vengavasi et al. (2017), who found that low-phosphorus stress promoted the synthesis of MDH and PEPC in soybean roots. The results of studies on the synthesis of hydrogenase and PEPC are the same, but Fredeen et al. (1990); Liao et al. (2006), and Liang et al. (2013) reported that low-phosphorus stress promoted the accumulation of malate in soybean roots and reduced the activities of 3 enzymes in soybean leaves, namely, phosphoglycerate kinase, TPI, and 1,6-bisphosphate aldolase (FBA). Moreover, low-phosphorus stress, as reported by Vengavasi et al. (2017), inhibited pyruvate kinase activity and reduced the accumulation of alkenes in soybean roots. Studies have shown that the synthesis of alcohol dehydrogenase, phosphoglycerate kinase, and TPI differed. This may be because the long-term low-phosphorus stress in this study partially stimulated the activity of the enzyme that converts glyceraldehyde-3-phosphate to malate but inhibited the synthesis of malate, while malate dehydrogenase, a bidirectional enzyme, converts malic acid to oxaloacetic acid, which is involved in amino acid metabolism (specifically the formation of aspartic acid, which is further converted to asparagine). Low-phosphorus stress accelerated glycolysis in soybean roots, which was accompanied by energy release, but inhibited malic acid synthesis, indicating that low-phosphorus stress promoted phosphorus transport in soybean roots.

### Regulation of phosphorus synthesis in soybean roots in response to low-phosphorus stress

**FIGURE 9:**
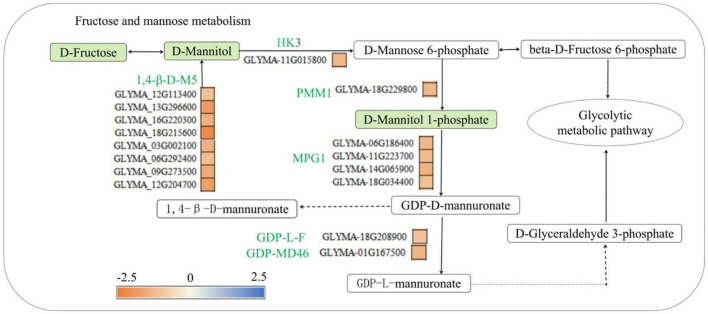
Fructose and mannose metabolic pathways involved in phosphorus synthesis in soybean roots. HK3, hexokinase 3, PMM1, phosphomannose mutase 1, MPG1, mannose-1-phosphoguanylate transferase, GM46D, GDP-mannose 4, 6 Dehydratase; GLF, GDP-L-fucose synthase; 1,4-β-D-M5, 1,4-β-mannosidase. Green solid squares indicate downregulated metabolites, red solid squares indicate upregulated metabolites. The red fonts represent upregulated genes, the green fonts represent downregulated genes, the red squares represent upregulated proteins, and the yellow and blue squares represent the fold change of gene expression.

Leguminous plants absorb phosphorus through their root hairs and epidermis, and 85% of the phosphorus in plants is involved in the synthesis of phosphorus-containing compounds, such as fructose-6-phosphate, mannose-1-phosphate and mannose-6-phosphate (Fredeen et al., 1990). Mannose can also be phosphorylated to mannose-6-phosphate (Freshour et al., 2003), the process of which is catalyzed by glucokinase, and then, mannose-6-phosphate can by catalyzed by phosphomannose mutase (PMM) to generate D-mannose-1-phosphate. D-mannose-1-phosphate is then used for mannose synthesis and metabolism or is converted to fructose-6-phosphate, which can then enter the glycolysis pathway (Klimacek et al., 1999; Eastmond and Graham, 2003). This study found that low-phosphorus stress reduced the concentration of mannose, it is consistent with the physiological data in Supplementary Table 6, and mannose-1-phosphate in the roots of the P- side of soybean, and the expression of hexokinase (HK3) (*GLYMA-11G015800*), phosphomannose mutase (PMM1) (*GLYMA-18G229800*), mannose-1-phosphate guanylyl transferase (MPG1) (*GLYMA-06G186400, GLYMA-11G223700*, *GLYMA-14G065900*, *GLYMA-18G0034400*), GDP-mannose-4,6-dehydratase (GM46D) (*GLYMA-01G167500*), GDP-L-fucosidase (GLF) (*GLYMA-18G208900*), and 1,4-β-mannosidase (1,4-β-D-M5) (*GLYMA-12G113400*, *GLYMA-13G296600*, *GLYMA-16G220300*, *GLYMA-18G215600*, *GLYMA-03G002100*, *GLYMA-06G292400*, *GLYMA-09G273500*, and *GLYMA-12G204700*) was downregulated (Figure 9). This is similar to the results of Raghothama and Karthikeyan. (2005) who found that low-phosphorus stress reduced the activity of phospho mannitol isomerase, inhibited the synthesis and use of mannose-6-phosphate by glycolysis, and, then, affected the transport of phosphorus in plants. Low-phosphorus stress inhibited the synthesis of fructose, mannose and mannose-1-phosphate and the synthesis of several phosphorus transport-related enzymes and invertase, indicating that low-phosphorus stress inhibited the transport and synthesis of several organic phosphorus-containing compounds.

## Conclusion

Low-phosphorus stress promoted the uptake of phosphorus by soybean roots and the transport of phosphorus from the P+ side to the P- side, and low-phosphorus stress regulated the metabolic pathways involved in starch and sucrose metabolism, glycolysis metabolism, fructose and mannose metabolism, etc., thereby affecting phosphorus transport in soybean roots. Low-phosphorus stress inhibited fructose synthesis and sucrose synthase synthesis in soybean roots and the synthesis of HK and fructose kinase, which converts fructose to fructose-6-phosphate. Low-phosphorus stress also promoted the synthesis of sucrose invertase and the conversion of sucrose into maltose, which is catalyzed by StS, and stimulated the synthesis of UGP and GP1 from UDP-glucose through the conversion of glucose-6-phosphate. The phosphorus transport pathway of the soybean roots was then affected, which promoted the incorporation of phosphorus into UTP and glucose-6-phosphate. Additionally, low-phosphorus stress accelerated glycolysis in soybean roots and inhibited the synthesis of malic acid, thereby promoting the transport of phosphorus in soybean roots. Low-phosphorus stress also inhibited the synthesis of fructose, mannose and mannose-1-phosphate and the synthesis of several enzymes involved in phosphorus transport as well as invertase, thereby inhibiting the transport and synthesis of several organic phosphorus-containing compounds.

## Data availability statement

Data for our manuscripts are published in the following databases https://www.ncbi.nlm.nih.gov/sra/PRJNA866385 accession number is PRJNA866385 and http://www.ebi.ac.uk/pride accession number is PXD035894.

## Author contributions

HL and CM contributed to the conceptualization and carried out the design of this research work. LX and JL carried out the experiment. CY and CW analyzed the data. HL wrote the manuscript. XL, XW, and SL supervised and complemented the writing of the manuscript. All authors read and approved the final manuscript.
