# Supplementary material for: Multi-omics analysis of the regulatory effects of low-phosphorus stress on phosphorus transport in soybean roots
Source: Front Plant Sci. 2022 Sep 2;13:992036. doi: 10.3389/fpls.2022.992036 (PMC9478169; doi:10.3389/fpls.2022.992036)
Supplement: Supplementary file 1 [file Data_Sheet_1.zip › Methods S1.doc]

S1 Preparation of Plant Materials with a Dual Root System:

Potted cultivation with river sand was used in this study. Each plastic pots was 0.30 m in diameter and 0.28 m in height. Each was divided into two equal parts in the middle with a custom-made polycarbonate plastic plate that fit the inside of the pot well. The top of the plastic plate was 2 cm lower than the edge of the pot, and the gaps between the pot and the partition plate were all sealed with glue. Two drainage holes 1 cm in diameter were drilled at the bottom of the pot, with one on each side of the partition plate.

Each pot was filled with 20 kg of river sand. Before use, the sand was thoroughly washed with tap water and then rinsed twice with distilled water.

Dual Root System：

The soybean seeds were sown in the fine-sand medium at a depth of 2 cm and cultured in an incubator at 30°C for approximately 3 days. When the distance between the growing point of the cotyledon and the tip of root reached approximately 7-10 cm, the root system of soybean seedlings was exposed by rinsing with distilled water.

First, a 0.5-1.0-cm-long incision (the seedling was not severed) was made upward with a sterilized blade from the root to the cotyledon at the site above the middle point of the hypocotyls in one soybean seedling. In the other seedling, a similar slash was made downward from the cotyledon to the root.

After the Two seedlings were cross-inserted into the cuts of each other, the grafting site was fixed with a grafting clip.

Two seedlings were separately planted into fine-sand medium of each half of the pot divided by the partition plate.

After a week, the grafting clip was removed. In one seedling, the top part above the grafting site was cut, leaving only the grafting site and the lower part. As a result, a seedling containing two root systems and one aboveground part was obtained. Two successfully grafted soybean seedlings were left in each pot to generate a one-plant/two-root-system soybean.
