# Supplementary material for: Multi-omics analysis of the regulatory effects of low-phosphorus stress on phosphorus transport in soybean roots
Source: Front Plant Sci. 2022 Sep 2;13:992036. doi: 10.3389/fpls.2022.992036 (PMC9478169; doi:10.3389/fpls.2022.992036)
Supplement: Supplementary file 1 [file Data_Sheet_1.zip › Methods S2.doc]

Detailed detection parameters of

*1 Proteomic analysis*

Protein extraction: One gram of sample tissue was added to liquid nitrogen for grinding, and the proteins were extracted by using SDT (4% w/v SDS, 100 mM Tris/HCl (pH 7.6), 0.1 M dithiothreitol [DTT]) buffer, and then the protein was quantified by a BCA Protein Assay Kit (Bio-Rad, USA). The proteins from each sample were digested by trypsinization via a filter-assisted proteome preparation kit. The peptide was desalted through C18 cartridges and redissolved in 40 μL of 0.1% formic acid solution after lyophilization. Finally, the peptides were quantified by determining the optical density at 280 nm (OD280).

Tandem mass tag (TMT) labeling and protein digestion: According to the manufacturer ' s instructions ( Thermo Fisher Scientific, USA ), TMT reagent was used to label 1×10^^(-4)^ g peptide mixture for each sample. The labeled peptides were fractionated by a High-pH Reversed-Phase Peptide Fractionation Kit (Thermo Fisher Scientific, USA) by gradually increasing the gradient elution with acetonitrile.

Fractionation was performed as follows. A high-pH reversed-phase peptide fractionation kit (Thermo Scientific) was applied to fractionate labeled peptides. After mixing and lyophilization, 100 μg of tagged peptide sample was diluted with 300 µL of trifluoroacetic acid (TFA) and transferred to a high-pH RP spin column. (a) The protective head of the column was removed and placed in a 2.0-mL centrifuge tube, which was centrifuged at 5000 ×g for 2 min to remove the original reserve liquid in the column. (b) Then, 300 μL of acetonitrile (ACN) was added to the column, which was placed in a 2.0-mL centrifuge tube, followed by centrifugation at 5000 ×g for 2 min; this step was repeated once. (c) Then, 300 μL 0.1% TFA was added to the column, which was placed in a 2.0-mL centrifuge tube and centrifuged at 5000 ×g for 2 min; this step was repeated once. (d) The column was added to a new 2.0-mL centrifuge tube and centrifuged at 3000 ×g for 2 min. (e) Then 300 μL water was added to the column, followed by centrifugation at 3000 ×g for 2 min in a new 2.0-mL centrifuge tube. (f) The 2.0-mL centrifuge tube was replaced with a new tube, and 300 μL of different dilutions of the eluent was used to elute the samples from the column (8%, 10%, 11.5%, 13%, 14.5%, 16%, 17.5%, 19%, 22%, and 50% ACN in 0.1% triethylamine) by centrifuging at 3000 ×g for 2 min, collecting the eluent as F1-F10, respectively. The collected fractions were desalted on C18 cartridges and concentrated by vacuum centrifugation. After vacuum-drying, the freeze-dried samples were redissolved in 12 μL of 0.1% FA and quantified by measuring the OD280.

Liquid chromatography–tandem mass spectroscopy (LC–MS/MS) analysis was performed on a Q Exactive Mass Spectrometer (Thermo Scientific) that was coupled to Easy nLC instrument (Proxeon Biosystems, now Thermo Fisher Scientific) for 60/90 minutes. The peptides were loaded onto a reversed-phase trap column (Thermo Scientific Acclaim PepMap100, 100 μm*2 cm, nanoViper C18) connected to a C18 reversed-phase analytical Easy Column (10 cm long, 75 μm inner diameter, 3 μm resin; Thermo Scientific) in buffer A (0.1% formic acid) and separated with a linear gradient of buffer B (84% acetonitrile and 0.1% formic acid) at a flow rate of 300 nl/min controlled by IntelliFlow technology, as described by Wang et al. (2020). After chromatographic separation, the sample was analyzed on a Q Exactive Mass Spectrometer in positive ion mode.

Proteome Discoverer 1.4 software with the MASCOT engine (version 2.2; Matrix Science, London, UK) was used for the identification and quantitation of proteins using the data for “Glycine_max” from the NCBI database.

*2 Metabolomic analysis*

Metabolite extraction: After the sample was slowly thawed at 4 °C, 50 mg was weighed into a precooled of methanol:acetonitrile:water mixture (2:2:1, v/v), vortexed, sonicated at low temperature for 30 minutes, and maintained at -20 °C. The mixture was incubated at room temperature for 10 minutes and subsequently centrifuged at 14000 × g for 20 minutes at 4 °C. The supernatant was collected and dried under a vacuum. Then, 100 μL of acetonitrile aqueous solution (acetonitrile:water = 1:1, v/v) was added for mass spectrometry analysis, reconstituted, and vortexed at 4 °C. The mixture was subsequently centrifuged at 14000 × g for 15 minutes, and the supernatant was collected for injection and analysis according to the methods of Wang et al. (2022).

LC-MS analysis:Samples were separated by an Agilent 1290 Infinity LC ultra-high-performance liquid chromatography (UHPLC) HILIC column. The column temperature was 25 °C, the flow rate was 0.5 mL/min, and the injection volume was 2 μL. The mobile phase solution A consisted of water + 25 mM ammonium acetate + 25 mM ammonia; B, acetonitrile. The gradient elution program was as follows: 0-0.5 minutes, 95% B; 0.5-7 minutes, linear decrease in B from 95% to 65%; 7-8 minutes, linear decrease in B from 65% to 40%; 8-9 minutes, maintenance of B at 40%; 9-9.1 minutes, linear increase in B from 40% to 95%; and 9.1-12 minutes, maintenance of B at 95%. The samples were placed in a 4°C autosampler throughout the analysis. For quadrupole time-of-flight–mass spectrometry (Q-ToF-MS), an AB Triple TOF 6600 mass spectrometer was used for the acquisition of first- and second-stage mass spectra. With respect to the electrospray ionization (ESI) source after separation by HILIC,the conditions were as follows. The ion source gas 1 (Gas1) was set at 60, ion source gas 2 (Gas2) was set at 60, curtain gas (CUR) was set at 30, the source temperature was set at 600℃, IonSpray Voltage Floating (ISVF) was set at ±5,500 V in positive and negative modes, the TOF MS scan m/z range was set from 60-1,000 Da, the product ion scan m/z range was set from 25-1,000 Da, the TOF MS scan accumulation time was set at 0.20 s/spectrum, and the product ion scan accumulation time was set at 0.05 s/spectra. Second-stage mass scans were obtained by information-dependent acquisition (IDA) in high-sensitivity mode with a declustering potential (DP) of ±60 V (positive and negative modes) and a collision energy of 35±15 eV. The settings for IDA were “Exclude Isotopes within 4 Da” and “Candidate ions to monitor per cycle: 10”.

**Statistical analysis**

Transcriptome analysis: The original image data files obtained during high-throughput sequencing were converted into original sequences (sequenced reads) by CASAVA base calling (base calling) analysis, stored in FASTQ (referred to as fq) file format, and then subjected to Gene Ontology (GO) annotation analysis and Kyoto Encyclopedia of Genes and Genomes (KEGG) annotation analysis. The GO annotation information refers to the GO (http://www.geneontology.org) functional classification system, and the KEGG annotation information refers to the KEGG (http://www.kegg.jp) system. Differentially expressed genes were screened according to the criteria padj < 0.05 and |log2(fold-change [FC]) greater than 1.

Proteomic analysis: The basic data from mass spectrometry were input into Proteome Discoverer 1.4 and MASCOT (Matrix Science, London, UK; version 2.2) for library term identification and quantitative analysis. By the use of NCBI BLAST + client software (ncbi-blast-2.2.28 + -win32.exe), an InterProScan search was performed by the use of Blast2GO to determine the GO functional enrichment of the target proteins. The KEGG pathway database was used to analyze metabolic pathways enriched in DEPs. The screening criteria for DEPs included an expression FC of more than 1.2 or less than 0.83 and a P value < 0.05 (Student’s T test).

Metabolomic analysis: The basic data were converted into mzXML format by ProteoWizard in Wiff format, and then peak alignment, retention time correction and peak area extraction were performed by XCMS software. The data extracted by XCMS were subjected to metabolite structure identification, data preprocessing, experimental data quality evaluation, and, finally, analysis. SIMCA-PV 15.0.2 software was used for principal component analysis (PCA) and orthogonal projections to latent structures-discriminant analysis (OPLS-DA), and an OPLS-DA variable importance in projection (VIP)>1 and a P value < 0.05 were used as criteria to screen significantly differentially expressed metabolites (DEMs). In addition, Student's t test and fold variation analysis were performed, and volcano plots were constructed by R software. Pathway annotations of the DEMs was performed using the KEGG database (http://www.kegg.jp/).

Multi-omics combined data analysis: Based on the KEGG annotation data, Fisher’s exact test and the respective differentially accumulates molecules revealed by the three different omic analysis procedures were used for KEGG pathway enrichment analysis.
