# Supplementary material for: Multi-omics analysis of the regulatory effects of low-phosphorus stress on phosphorus transport in soybean roots
Source: Front Plant Sci. 2022 Sep 2;13:992036. doi: 10.3389/fpls.2022.992036 (PMC9478169; doi:10.3389/fpls.2022.992036)
Supplement: Supplementary file 1 [file Data_Sheet_1.zip › Table S1.doc]

**Table S1.**  Composition of sand culture nutrient solution (mg/L)

| inorganic salts | Concentration(mg/L) | inorganic salts | Concentration(mg/L) |
| --- | --- | --- | --- |
| （NH_4_)_2_SO_4_ | 235.80 | ZnSO_4_·7H_2_O | 0.22 |
| MgSO_4_ | 240.00 | MnCl_2_·4H_2_O | 4.90 |
| CaCl_2_ | 220.00 | H_3_BO_3_ | 2.86 |
| Na_2_MoO_4_·H_2_O | 0.03 | Fe–EDTA* | * |
| CuSO_4_·5H_2_O | 0.08 | — | — |

* Dissolve 5.57g of FeSO_4_•7H_2_O and 7.45g of Na_2_EDTA to 1L respectively, and add 1ml of stock solution per liter of nutrient solution when using.
