# Supplementary material for: Multi-omics analysis of the regulatory effects of low-phosphorus stress on phosphorus transport in soybean roots
Source: Front Plant Sci. 2022 Sep 2;13:992036. doi: 10.3389/fpls.2022.992036 (PMC9478169; doi:10.3389/fpls.2022.992036)
Supplement: Supplementary file 1 [file Data_Sheet_1.zip › Table S2.doc]

**Table S2. The elements of P or K in different P level treatments**

| treatment | inorganic salts concentration( mg/L) | | | |
| --- | --- | --- | --- | --- |
|  | KH_2_PO_4_ | K_2_SO_4_ | KCl | NaH_2_PO_4_·2H_2_O |
| P1 | 4.39 | 42 | 36 | — |
| P31 | 136 | — | — | — |
| P0 |  | 43.5 | 37.25 |  |
