# Supplementary material for: Multi-omics analysis of the regulatory effects of low-phosphorus stress on phosphorus transport in soybean roots
Source: Front Plant Sci. 2022 Sep 2;13:992036. doi: 10.3389/fpls.2022.992036 (PMC9478169; doi:10.3389/fpls.2022.992036)
Supplement: Supplementary file 1 [file Data_Sheet_1.zip › Table S6.doc]

| **Table S6.**Key metabolites content ofphosphorus-free side of dual-root soybean roots | | | | |
| --- | --- | --- | --- | --- |
| Treatments | Malic acid  (μg/g FW） | asparagine  (μg/g FW） | mannose  （mg/g FW) | maltose  （mg/g FW) |
| LP | 864±13.89 b | 31.28±13.93 b | 0.90±0.02 b | 1.8±0.02 a |
| OP | 1110±57.94 a | 26.87±9.32 b | 1.6±0.07 a | 1.1±0.01 b |

The data are represented as the mean values ± standard error and independent measurements with four replicates. Different lowercase letters indicate a significant difference between the treatments at the 5% level.
